# Cabozantinib Affects Osteosarcoma Growth Through A Direct Effect On Tumor Cells and Modifications In Bone Microenvironment

**DOI:** 10.1038/s41598-018-22469-5

**Published:** 2018-03-08

**Authors:** M. Fioramonti, V. Fausti, F. Pantano, M. Iuliani, G. Ribelli, F. Lotti, Y. Pignochino, G. Grignani, D. Santini, G. Tonini, B. Vincenzi

**Affiliations:** 1 Medical Oncology, Campus Bio-Medico University of Rome, Rome, Italy; 2 Medical Oncology, University of Ferrara, Ferrara, Italy; 3 Osteoncology and Rare Tumors Center, Istituto Scientifico Romagnolo per lo Studio e la Cura dei Tumori (IRST) IRCCS, Meldola (FC), Italy; 4 Orthopaedics and Trauma Surgery, Campus Bio-Medico University of Rome, Rome, Italy; 5 Sarcoma Unit, Division of Medical Oncology, Candiolo Cancer Institute-FPO, IRCCS, Candiolo (TO), Italy

## Abstract

Osteosarcoma (OS) is the most common primary malignant tumor of the bone. Due to its high heterogeneity and to survival signals from bone microenvironment, OS can resist to standard treatments, therefore novel therapies are needed. c-MET oncogene, a tyrosine-kinase receptor, plays a crucial role in OS initiation and progression. The present study aimed to evaluate the effect of c-MET inhibitor cabozantinib (CBZ) on OS both directly and through its action on bone microenvironment. We tested different doses of CBZ in *in vitro* models of OS alone or in co-culture with bone cells in order to reproduce OS-tumor microenvironment interactions. CBZ is able to decrease proliferation and migration of OS cells, inhibiting ERK and AKT signaling pathways. Furthermore, CBZ leads to the inhibition of the proliferation of OS cells expressing receptor activator of nuclear factor κB (RANK), due to its effect on bone microenvironment, where it causes an overproduction of osteoprotegerin and a decrease of production of RANK ligand by osteoblasts. Overall, our data demonstrate that CBZ might represent a new potential treatment against OS, affecting both OS cells and their microenvironment. In this scenario, RANK expression in OS cells could represent a predictive factor of better response to CBZ treatment.

## Introduction

Osteosarcoma (OS) represents the most common primary malignant tumor of the bone and it affects children and adolescents with a second peak in incidence in adults over the age of 50^1,2^.

Currently, main therapies include surgical resection and combinational chemotherapy (doxorubicin, cisplatin with methotrexate). The EURAMOS trial (NCT00134030) is one of the most important clinical trial involving 2260 OS patients that aimed to assess the best therapy for OS^3^. Authors compared post-surgical methotrexate, doxorubicin, and cisplatin (MAP) plus interferon-α-2b (IFN-2b) versus MAP alone treatments (for deeper details about eligibility criteria, randomization and treatment procedures see references^3^ and^4^). In particular, the effect of the IFN-2b treatment in the whole population was estimated as hazard ratio (HR) of 0.83 (95% CI, 0.61 to 1.12; P = 0.214), whereas the rates of 3-year effect-free survival (EFS) were 74% (95% CI, 69% to 79%) and 77% (95% CI, 72% to 81%), for MAP and MAP plus IFN-2b, respectively. Neither OS patients subgroup with localized disease showed significant improvement if treated with IFN-2b (HR = 0.83; 95% CI, 0.59 to 1.17; P = 0 .284). Thus, no significant differences were found between the two treated arms, confirming that standard chemotherapy is still the best treatment for OS^4^. Even if these therapies result in long-term survival rates of 60% to 70% in patients with localized disease, patients with metastatic or relapsed OS have an overall 5-year survival rate of about 20%^2,5^. Thus, alternative therapies able to improve clinical outcome in OS patients are needed.

Several therapeutically targetable tyrosine kinase receptors or their ligands are overexpressed in OS, including KIT, Vascular endothelial growth factor receptor (VEGFR) -2, -3, Platelet derived growth factor (PDGFR)-β and MET^6,7^. This overexpression correlates with metastasis onset and poor survival in patients with OS^5^. Starting from these evidences, in the last few years several targeted therapies have been investigated.

For instance, sorafenib, an inhibitor of RAF, VEGFR-2/3, FLT-3, KIT, FGFR-1, RET, MCL-1 and PDGFR-β, reduces proliferation and induces apoptosis in OS cell lines^7^. Moreover, the combination of sorafenib and everolimus, an inhibitor of mammalian target of rapamycin (mTOR), enhanced antiproliferative, proapoptotic and antiangiogenic effects, reducing tumor growth and its propensity to metastasize in OS mice model^8^. Another multi-kinase inhibitor sunitinib, an anti-PDGFRα/β, VEGFR1/2/3, KIT, FLT3, CSF- b1R and RET, has been shown to decrease primary tumor proliferation and reduce tumor vasculature in cell-derived intratibial OS model in SCID mice^9^. Many other details about the effect of novel targeted therapies on OS are exhaustively analyzed by Kansara and colleagues in their review^2^.

Unfortunately, multikinase inhibitors showed only limited efficacy in advanced OS because of its high heterogeneity in terms of disease-driving genetic aberrations^10^. Conversely, OS microenvironment, in particular bone cells (i.e. osteoblasts and osteoclasts), due to its more homogenous physiology, may represent a more suitable therapeutic target. Moreover, it has been demonstrated that bone microenvironment communicates and interacts with OS cells, playing a key role in growth, metastasis and cancer stem cell fate^11^. Indeed, some studies showed that the receptor activator of nuclear factor κB (RANK) is expressed by human OS cells^12^ and represents a negative prognostic factor in terms of disease-free survival in OS patients^13^. In this scenario, osteoblasts (OBLs) are the most important source of RANK ligand (RANKL) within the bone microenvironment and, therefore, could exert a pro-tumorigenic effect on RANK-positive osteosarcoma cells stimulating their growth and proliferation^12,13^.

These evidences led to a paradigm shift in therapeutic strategy from approaches primarily directed against tumor cells to a development of novel molecules able to target also tumor microenvironment.

From this point of view, cabozantinib (CBZ) is a novel inhibitor of multiple tyrosine kinase receptors, (including c-MET, VEGFR-2. Ret, Kit, Flt-1/3/4, Tie2, and AXL), showing a strong efficacy against several type of tumors such as thyroid cancer, renal cancer and prostate cancer^14–16^. It has been demonstrated that c-MET, one of the main targets of CBZ, is overexpressed in OS. Moreover, overexpression of c-MET in primary human OBLs resulted in their transformation into OS cells, displaying most of the distinguishing features of primary OS^17,18^. c-MET inhibition strategy against OS has been already tested; indeed, crizotinib, a potent inhibitor of c-Met and ALK, showed encouraging results to inhibit malignant properties of OS cells *in vitro* and orthotopic xenograft growth *in vivo*^19^. Unlike crizotinib, there are several studies that demonstrated a strong impact of CBZ on bone microenvironment, both in murine and human models^20–22^. In particular, our group has previously demonstrated that CBZ has a strong impact on bone microenvironment, inducing a decrease of RANKL secretion and stimulating the overproduction of osteoprotegerin (OPG), a soluble receptor of RANKL, in human primary OBLs^23^.

Taking into account the importance of c-MET pathway in OS and the impact of CBZ on bone microenvironment, the aim of this study was to analyze the direct effect of CBZ treatment on preclinical *in vitro* model of OS. Moreover, we investigated CBZ potential “indirect” anti-tumorigenic effect mediated by bone microenvironment using a cell-to-cell contact co-culture model between OS cells and OBLs.

## Results

### OS cell lines functionally express the c-MET receptor

**Figure 1:**
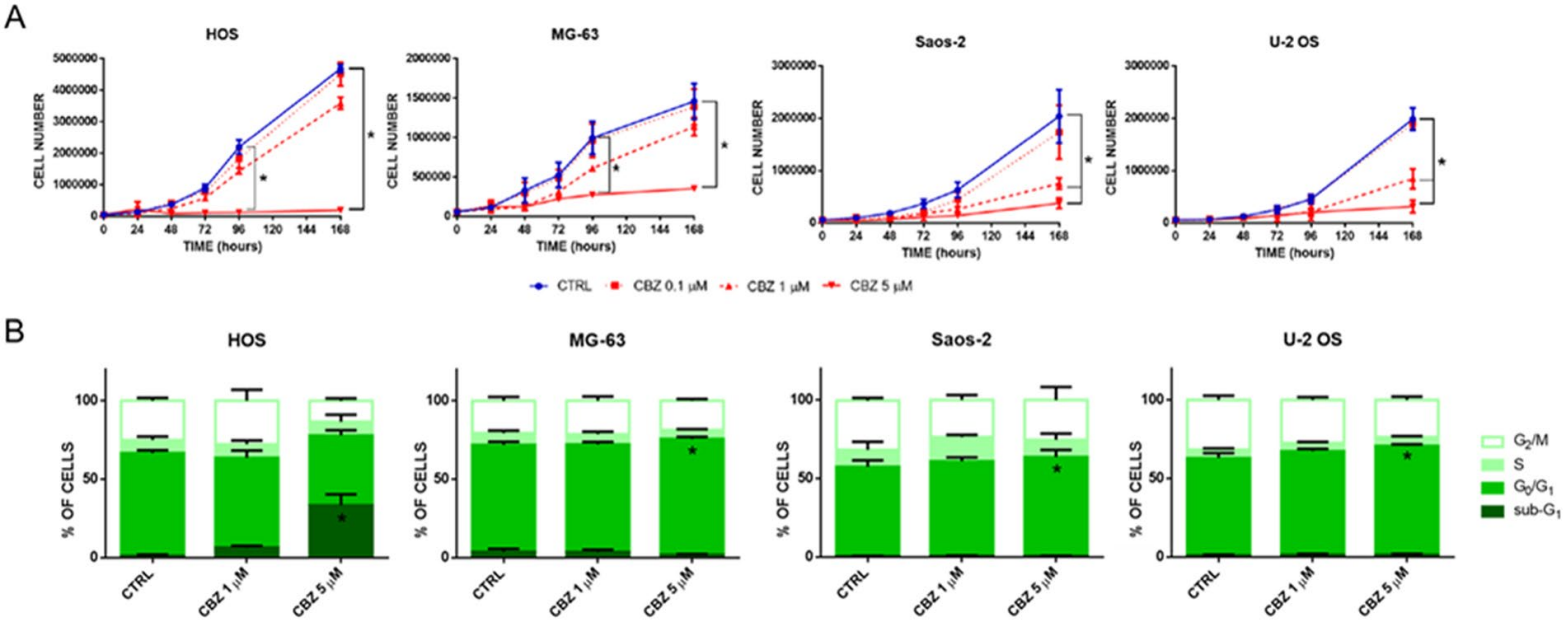
Evaluation of the impact of CBZ treatment on HOS, MG-63, Saos-2 and U-2 OS cells proliferation. (**A**) Proliferation curves of OS cells treated with different doses of CBZ or DMSO as control; CBZ 5 μM significantly inhibits the proliferation of each OS tested cell line. (**B**) Cell cycle analysis of OS cells treated with different doses of CBZ or DMSO as control; CBZ 5 μM causes a little but significant block of cells in the G0/G1 phase of cell cycle in MG-63, Saos-2 and U-2 OS cells, but not in HOS cells where it induces a strong accumulation of cells in the sub-G1 phase.

OS cell lines were tested for c-MET expression by western blot. Each cell line strongly expresses the c-MET receptor at the same level (Fig. 1 supplementary data). In particular, HOS, MG-63 and Saos-2 cells exhibit high levels of both pro-MET at ~175 KDa and the active form of receptor c-MET at ~140 KDa. On the contrary, U-2 OS cells express high levels of only the active form of receptor, without any expression of the inactive pro-MET form.

### CBZ shows a dose-dependent inhibition of OS cells proliferation

In order to explore the effect of CBZ on cell proliferation, cell growth curves were performed using OS cell lines treated with 0.1, 1 and 5 μM of CBZ. This treatment shows a dose-dependent inhibition of proliferation rate in each cell line tested (Fig. 1A). In particular, CBZ 5 μM represents the most effective dose able to block the proliferation of OS cells. HOS and MG-63 cell lines proliferations are significantly inhibited by CBZ 5 μM after 96 hours of treatment (P = 0.0003 e 0.004, respectively). This inhibition remains significant after 168 hours of treatment (P = 0.0001 e 0.0006, respectively). Both 1 and 5 μM are able to inhibit the proliferation of Saos-2 and U-2 OS cell lines, but only at 168 hours of treatment (Saos-2 P-values = 0.02 and 0.005, respectively and U-2 OS P-values = 0.004 and 0.0009, respectively).

### CBZ induces a block of cell cycle progression in OS cells

To characterize the effect of CBZ on cell proliferation, OS cells treated with different doses of CBZ were analyzed for cell cycle progression by flow cytometer. MG-63, Saos-2 and U-2 OS cells showed a little but significant accumulation in the G_0_/G_1_ phase of cell cycle (P = 0.04, 0.03 e 0.03, respectively) after treatment with 5 μM CBZ (Fig. 1B). Conversely, 5 μM CBZ treated HOS cells showed a strong increase of the sub-G_0_ fraction, usually related to DNA fragmentation (Fig. 1B). Flow cytometric analysis of side scatter (SSC) and forward scatter (FSC) of OS cells treated with CBZ revealed that HOS cells, but not other OS cell lines, change their morphology and volume compared to control cells (data not shown).

### CBZ causes the formation of aberrant mitosis in HOS cells but not in other OS cell lines

**Figure 2:**
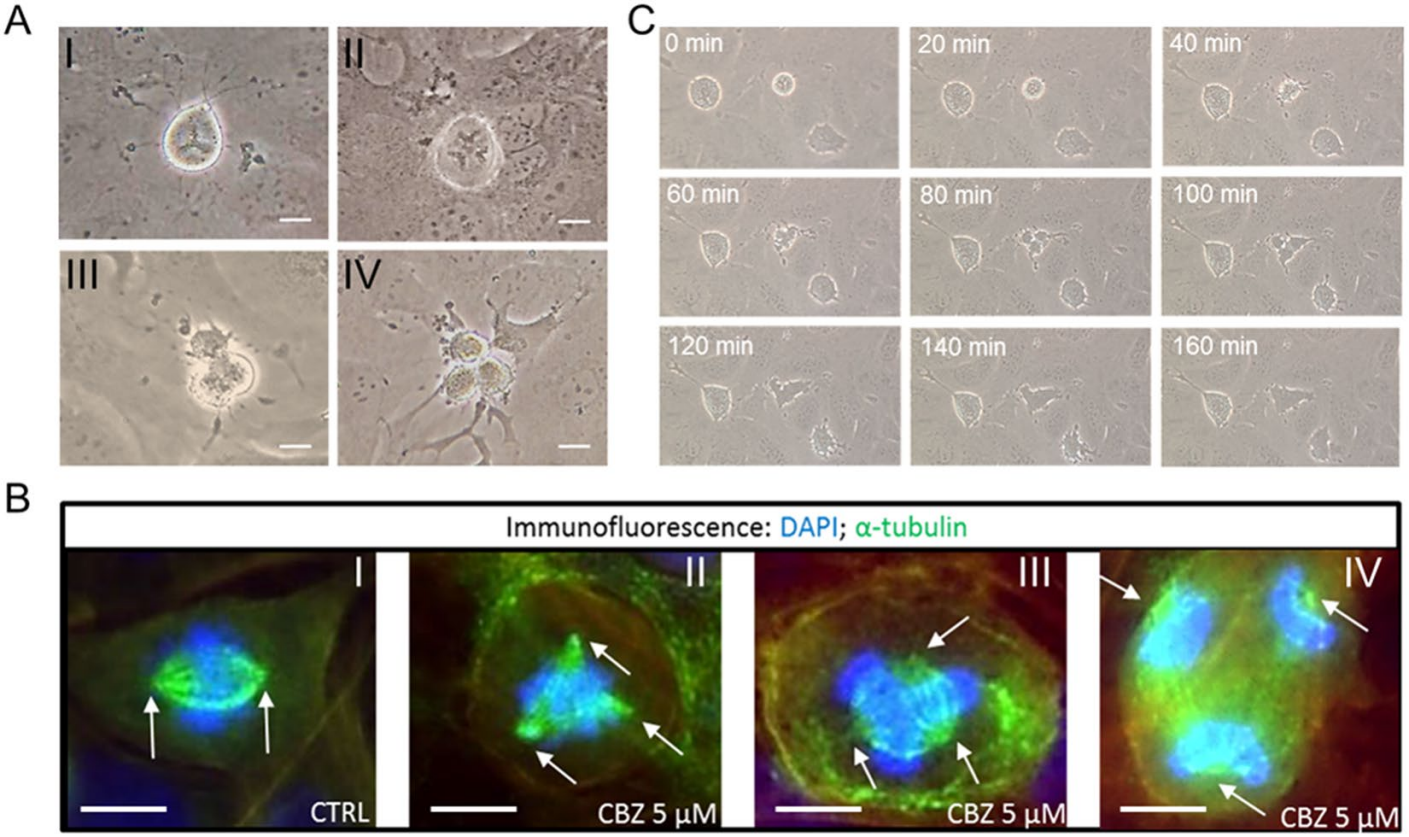
Analysis of HOS cells division. (**A**) CBZ 5 μM treatment leads to the formation of triple metaphases (I), triple anaphases (II), triple telophases (III) and triple cytodieresis (IV); bar scale = 20 μm. (**B**) immunostaining of mitotic spindle in control and CBZ 5 μM treated HOS cells using a monoclonal antibody against α-tubulin (in green); white arrows indicate the mitotic spindle poles; control cells show normal bipolar spindles during metaphase formation, whereas treated cells show tripolar spindles during prometaphase (II), metaphase (III) and anaphase (IV); bar scale = 10 μm. (**C**) time lapse analysis of cell division of CBZ 5 μM treated HOS cells; after 80 minutes from metaphase formation, cells start cytodieresis but at 160 minutes they failed to finish this phase of mitosis, resulting in a unique giant cell.

In order to confirm and better understand the mechanism behind the morphology change, OS cells proliferation was analyzed under a phase contrast microscope after 5 μM CBZ treatment. Several aberrant mitoses appeared in HOS treated cells, but not in control HOS or in other cell lines, after 24 hours of treatment (Fig. 2). In particular, it is possible to recognize triple metaphases, triple anaphases, triple telophases and triple cytodieresis (Fig. 2A). This data was confirmed by immunofluorescent staining of mitotic spindles using a monoclonal antibody for α-tubulin (Fig. 2B). In particular, bipolar spindles were found in control HOS and both in control and treated MG-63, Saos-2 and U-2 OS cells, whereas triple and sometimes tetra spindles were found in 5 μM CBZ treated HOS cells (Fig. 2B). Time-lapse analysis of 5 μM CBZ treated HOS cells revealed that after the formation of triple mitosis, the dividing cells do not carry out the cytodieresis, and the resulting final cell is represented by a unique giant cell (Fig. 2C).

### CBZ decreases the migration rate of OS cells

**Figure 3:**
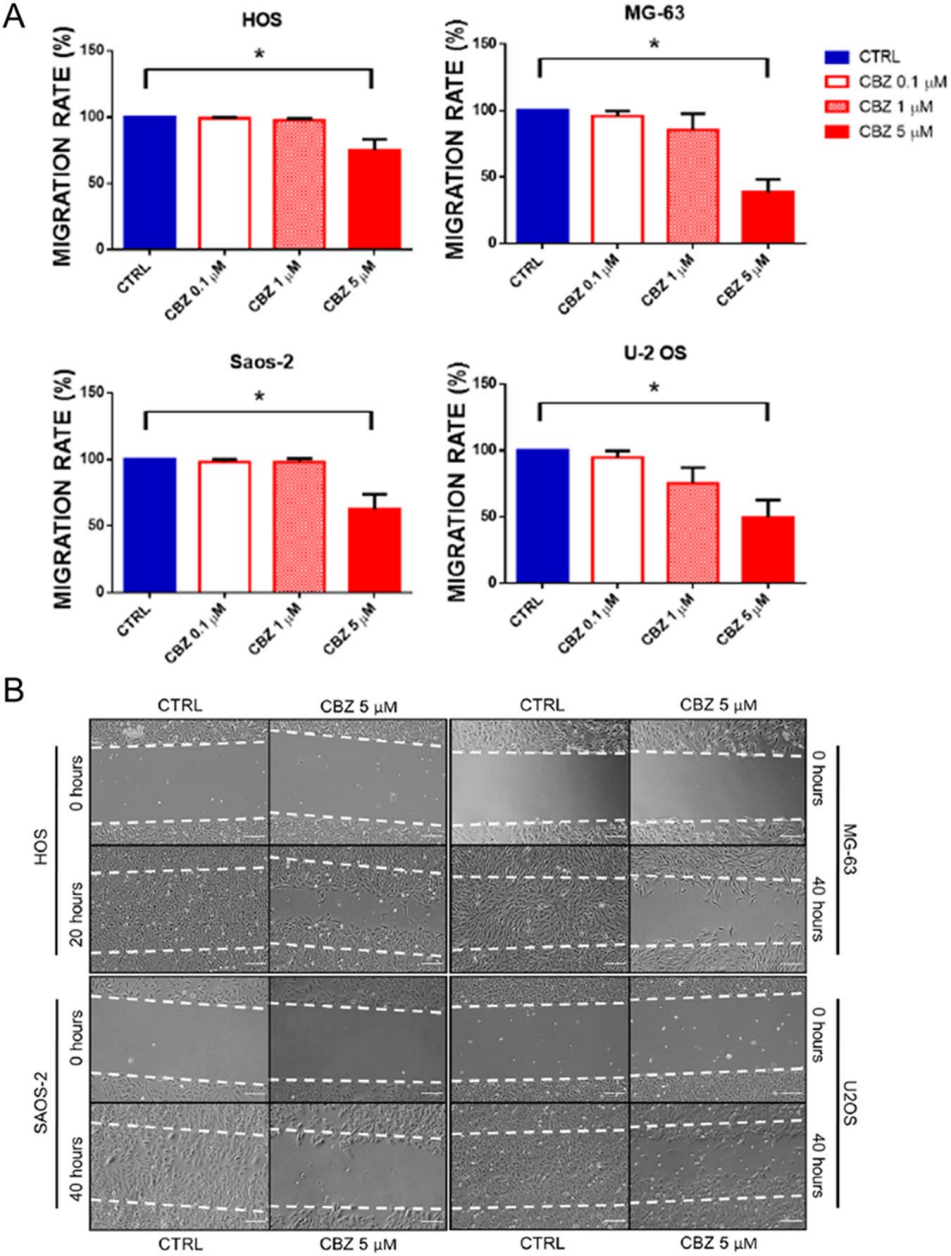
Evaluation of the impact of CBZ treatment on HOS, MG-63, Saos-2 and U-2 OS cells migration. (**A**) quantification of the migration rate in OS cells treated with different doses of CBZ or DMSO as control; CBZ 5 μM significantly decrease the migration rate of all OS cells tested; (**B**) representative images of wound healing assay on control and CBZ 5 μM treated cells; control HOS cells close the wound in 20 hours, whereas control MG-63, Saos-2 and U-2 OS in 40 hours; CBZ 5 μM treated OS cells can repair only a little percentage of the wound compared to their respective control cells.

Migration of OS cells treated with or without different doses of CBZ was evaluated using the wound healing assay. 5 μM CBZ shows a strong inhibition of migration in each tested OS cell line (Fig. 3). In particular, control HOS are able to close the wound after 20 hours, while 5 μM CBZ treated HOS cells cover only the 75% of area (P = 0.006). Control MG-63, Saos-2 and U-2 OS cells close the wound after 40 hours, while treated cells cover 38.5% (P = 0.0004), 62.2% (P = 0.007) and 49.4% (P = 0.002), respectively.

### CBZ blocks the activity of c-MET receptor and leads to an inhibition of ERK and AKT signalling pathways

**Figure 4:**
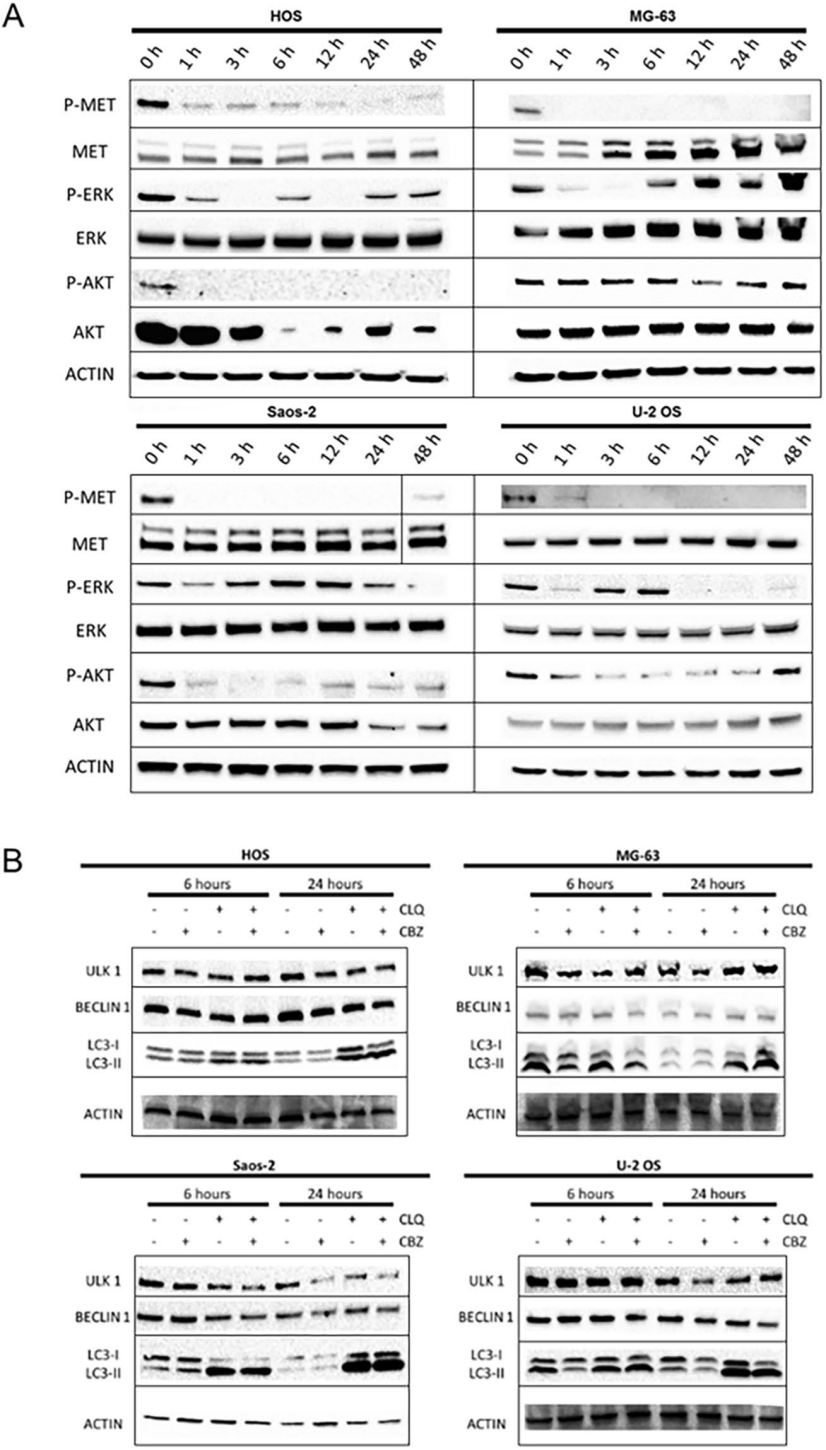
Western blot analysis of cellular pathways modifications after CBZ 5 μM treatment in HOS, MG-63, Saos-2 and U-2 OS cells; (**A**) CBZ 5 μM inhibits c-MET phosphorilation after 1 hour of treatment; downstream ERK activation kinetic adopts a sigmoidal shape in all OS cell lines, whereas AKT activation is strongly inhibited in HOS, Saos-2 and U-2OS, but not in MG-63 cells. (**B**) analysis of different markers of authophagy; CBZ 5 μM treated cells show an accumulation of active form of LC3 (LC3-II); the effect is more significant when OS cells are pre-treated with CLQ. Full-length blots are presented in Supplementary Figure 4.

In order to evaluate the effect of CBZ in the intracellular signaling pathways, OS cells were treated with 5 μM CBZ and proteins were extracted after 0, 1, 3, 6, 12, 24 and 48 hours. We showed that CBZ treatment leads to a potent decrease of the activity of c-MET receptor (evaluated as levels of c-MET protein phosphorylated in Tyr1234/1235) after 1 hour of treatment in each tested OS cell line (Fig. 4A). The activity of c-MET receptor remains inhibited after 48 hours of CBZ treatment in each cell line, but not in Saos-2, wherein normal phospho-MET levels are found after 48 hours. c-MET downstream pathways ERK and AKT were also tested. We observed sigmoidal waves of activation and inhibition of ERK protein (evaluated as levels of ERK protein phosphorylated in Thr202/Tyr204) with peculiar activation times in each OS cell line after CBZ treatment (Fig. 4A). Phospho-AKT levels (Thr308), indicating the activation of AKT pathway, were strongly decreased in each OS cell lines, except for MG-63 cells in which phospho-AKT protein level slightly decreased between 12 and 24 hours after treatment (Fig. 4A).

### CBZ treatment activates OS cells autophagy

Due to the deep alteration of intracellular pathways after CBZ treatment, OS cells could activate autophagy as a survival mechanism. For this reason, CBZ (5 μM) treated OS cells were tested for the activation of autophagy after 6 and 24 hours of treatment. ULK1, Beclin 1 and LC3-I/II were chosen as markers of autophagy. Samples were also pre-treated with Chloroquine (CLQ) 25 μM for 1 hour, in order to impair lysosomal acidification and maximize the possible effect of CBZ treatment on autophagy activation^24^. In each OS cell lines, there is an accumulation of the active form of LC3 after 24 of CBZ treatment (Fig. 4B). This effect was enhanced by the pre-treatment with CLQ, but it was not associated to an increase of ULK1 and Beclin 1, whose levels remained unchanged.

### CBZ pre-treated OBLs inhibit the proliferation of RANK-positive cell lines

Each OS cell line was tested for RANK expression by western blot. HOS, MG-63 and Saos-2 cells showed high levels of RANK receptor. Saos-2 cells expressed the highest level of RANK. Conversely, U-2 OS cells did not show any RANK signal (Fig. 2 supplementary data).

**Figure 5:**
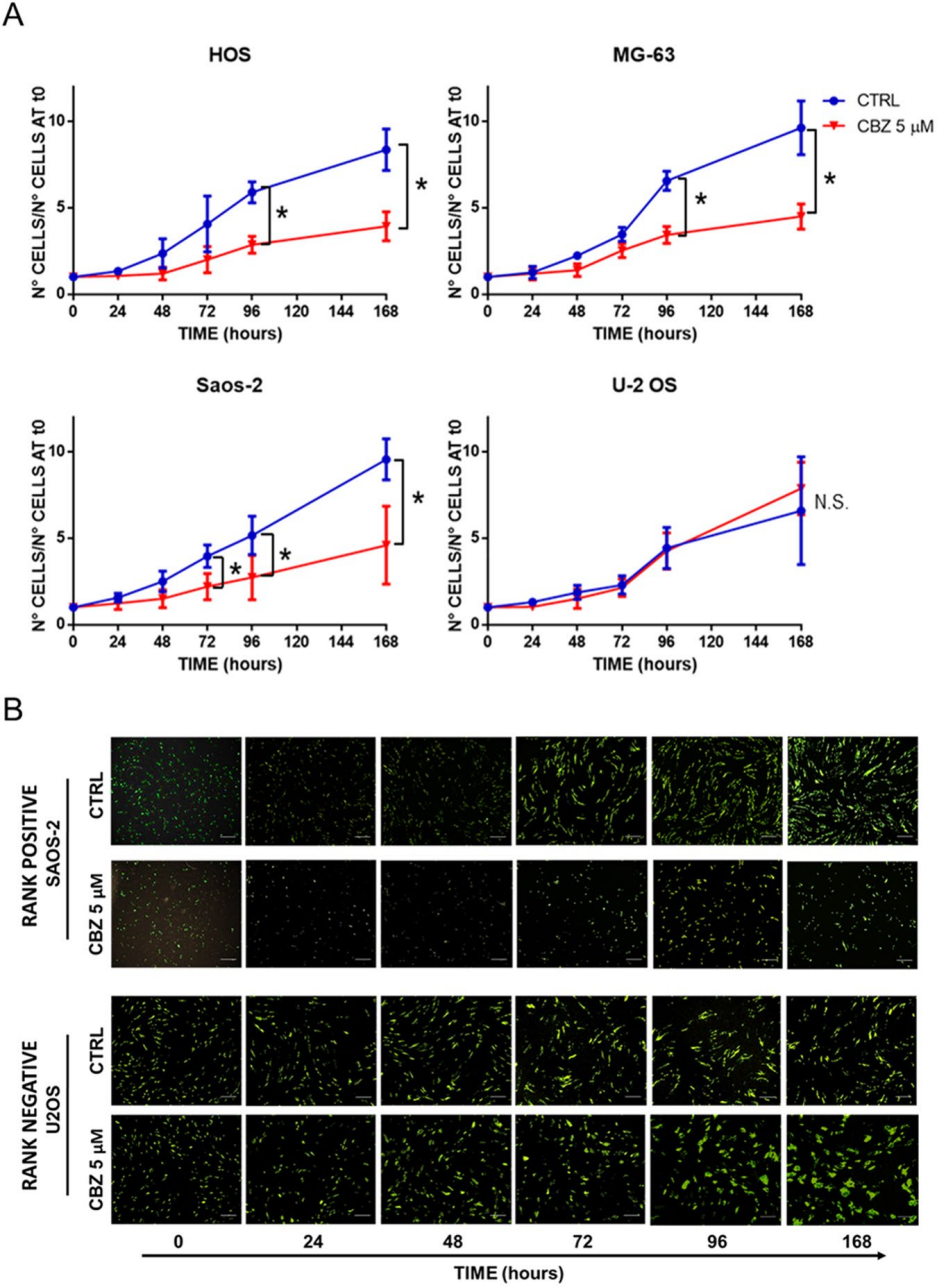
OBLs mediated effect of CBZ on HOS, MG-63, Saos-2 and U-2 OS cells. (**A**) Quantification of the proliferation rate of OS cells co-cultured with CBZ 5 μM or DMSO (as control) pre-treated OBLs; presence of CBZ 5 μM pre-treated OBLs leads to an inhibition of proliferation of RANK-positive HOS, MG-63 and Saos-2 cells, whereas no effect is detectable in U-2 OS cells. (**B**) representative images of proliferation curves between control or CBZ 5 μM pre-treated OBLs and RANK-positive Saos-2 or RANK-negative U-2 OS GFP + cells. Bar scale = 100 μm.

In order to evaluate the OBLs-mediated effect of CBZ on OS cells, we set up a direct co-culture experiment with 5 μM CBZ pre-treated or control OBLs and GFP + OS cells (Fig. 5A and B). The use of GFP + OS cells allowed us to discriminate between OS cells and OBLs (Fig. 3 supplementary data). Pre-treated OBLs were able to inhibit the proliferation of RANK-positive OS cells, but did not affect RANK-negative U-2OS cell line. In particular, the most RANK-expressing cell line Saos-2, showed a significant decrease of cell proliferation after 72 hours of co-culture with CBZ pre-treated OBLs compared to control (P = 0.02). This inhibition was significant also after 96 and 168 hours of co-culture (P = 0.04 and 0.03, respectively). HOS cells proliferation was significantly inhibited by the presence of CBZ pre-treated OBLs after 96 and 168 hours of co-culture (P = 0.04 and 0.03, respectively). Likewise, CBZ pre-treated OBLs strongly affected MG-63 proliferation after 96 and 168 hours of co-culture (P = 0.02 and 0.04, respectively). Conversely, the RANK-negative U-2 OS cell lines did not decrease the proliferation rate when co-cultured with CBZ pre-treated OBLs. Indeed, the presence of CBZ pre-treated OBLs seemed to lead to an increasing trend of U-2 OS cells growth that did not reach statistical significance.

## Discussion

c-MET is a transmembrane tyrosine kinase receptor overexpressed in several types of tumors that promotes cell survival, transformation, proliferation and motility^25^. Several studies have shown the crucial role of c-MET pathway in OS initiation and progression. c-MET receptor was discovered for the first time in an OS derived cell line by Cooper and colleagues in 1984^26^. The relevance of c-MET receptor in this tumor is demonstrated by the fact that OS represents the musculoskeletal tumor expressing the highest level of c-MET receptor^27^ and this overexpression in differentiated OBLs can drive their transformation into OS cells^17,18^. CBZ, a c-MET receptor inhibitor, has been demonstrated to improve progression free survival and reduce skeletal related event (SRE) in different tumor types such as thyroid cancer, castration resistant prostate cancer and, recently, metastatic renal carcinoma. Starting from these evidences, in the present study, we evaluated the anti-cancer effect of CBZ in cellular models of OS. Several studies evaluated the effect of CBZ on preclinical models of breast cancer, prostate cancer, renal cancer thyroid tumor and many others^28–30^, but there are no evidence about its effect on OS cells. Using four well-characterized OS cell lines, we firstly demonstrated their high expression of c-MET and then showed that 5 μM CBZ inhibits the receptor activity after 1 hour of treatment. The activity of c-MET receptor remains blocked after 48 hours, except for Saos-2 cells that can restore the normal level of c-MET activity after 48 hours. We found that the proliferation of OS cells was strongly affected by CBZ treatment in a dose-dependent manner. In particular, 5 μM CBZ led to a significant inhibition of OS cells proliferation after 96 or 168 hours of treatment. Lower doses of CBZ (1 μM) induced an inhibition of proliferation reaching statistical significance just in Saos-2 and U-2 OS cell lines. These results, in terms of cell growth reduction in OS, are comparable to those obtained with other drugs. For instance, methotrexate chemotherapy and two targeted therapies, sunitinib and sorafenib, are able to decrease proliferation of OS cells after 144 hours of treatment^7–9,31^. CBZ causes the inhibition of proliferation of MG-63, Saos-2 and U-2OS through a block of cells in the G_0_/G_1_ phase. The effect of CBZ on HOS cells proliferation is more intricate. The flow cytometry analysis in cell cycle of this cell line revealed a strong increase of cell volume coupled with a significant accumulation of CBZ treated cells in the sub-G_1_ fraction, usually related to apoptosis induction. Caspase-3 active protein levels (tested by western blot at the same time points of flow cytometry analysis) did not change in response to the treatment, showing no activation of apoptotic pathway (data not shown). Conversely, microscopic and time-lapse analysis of HOS cells showed that CBZ treatment caused the formation of aberrant mitosis characterized by triple functional spindle. Nevertheless, the dividing treated cells were not able to complete the cytodieresis leading to a unique cell with increased volume and aberrant amount of DNA, explaining the result of flow cytometry analysis. Since there are no literature evidences demonstrating similar effects of other drugs on OS proliferation, this could be a cell line specific effect. Further analyses are warranted to better elucidate this phenomenon.

In the present study, we also demonstrated that CBZ was able to inhibit OS cells migration, usually related to invasion and metastasis in tumor cells. In particular, the effect of CBZ (5 μM) on OS cells migration was similar to the effect of another c-MET inhibitor, SU11274, and other drugs such as inhibitors of EGFR, HER-2 and IGF-1R^5^. Since it has been demonstrated that Src pathway, downstream of the c-MET receptor^25^, is involved in OS cells migration^32^, we hypothesize that CBZ could inhibit migration through this signaling pathway. We also suggest that the result observed in the wound healing assay is only due to changing in the migration rate, and not to the decrease of the proliferation, because the massive effect appears in the early times of treatment.

We evaluated the effect of CBZ on two important intracellular pathways downstream of c-MET: ERK and AKT. ERK protein showed sigmoidal waves of inhibition and activity after CBZ treatment, probably due to some positive feedback mechanisms that try to restore the normal levels of ERK activity, but they do not succeed. AKT signaling is strongly inhibited by CBZ treatment in each tested OS cells, except for MG-63 cell line. These data could explain the strong inhibition of proliferation and migration due to CBZ treatment in OS cells. Indeed, ERK and AKT represent two of the most important intracellular pathways driving tumor cell survival, transformation and proliferation. It has been demonstrated that cancer cells are able to survive to metabolic and therapeutic stresses through autophagy^33^. Furthermore, AKT signaling is able to strongly influence the activation of autophagy. This emerging role suggested us to evaluate the possible induction of autophagy in response to CBZ treatment. The increased levels of LC3-II suggested that OS cells could activate the autophagic process to survive during CBZ treatment. This observation suggested to test combinations between anti autophagic drugs (e.g., CLQ) and CBZ to further increase the anti-tumor effect of c-MET inhibition.

CBZ treatment displayed a strong direct effect on OS cells affecting proliferation, migration and intracellular pathways. However these effects were not enough to arrest OS progression like other targeted therapies tested on OS cells^2,10^. We believe this short–lasting activity may be related to survival signaling from the bone microenvironment^11^ and, in particular, from OBLs, that could represent an innovative target. For this reason, a targeted therapy able to act not only on tumor cells, but also on bone microenvironment could be more effective for the treatment of OS. Our group has previously demonstrated that CBZ influences bone microenvironment^23^ leading to an overproduction of OPG and a decreased production of RANKL by OBLs (with a change of OPG/RANKL ratio from ~2.0 in ctrl OBLs to ~4.2 in CBZ-treated OBLs). Thus, the balance between OPG and RANKL is completely shifted in favor of the anti osteoclastogenic cytokine OPG, that is responsible of the decreased differentiation of osteoclasts (OCLs) during CBZ treatment. In this paper we evaluated if this strong change in bone microenvironment might affect the proliferation of OS cells. To be responsive to the RANKL/OPG ratio, OS cells have to express RANK receptor. Literature data demonstrated that ~60–65% of OS are RANK-positive and RANK expression represents a negative prognostic factor in OS patients^12,13^. As previously observed by Mori and colleagues^13^, we firstly demonstrated that HOS, MG-63 and Saos-2 cells are RANK-positive, whereas the U-2 OS cell line is RANK-negative. Thus, we set up a direct co-culture experiment with untreated (control) or CBZ pre-treated OBLs and GFP + OS cells. The presence of CBZ pre-treated OBLs strongly inhibited the proliferation of RANK-positive OS cells compared to control, whereas no significant decrease of proliferation was observed in RANK-negative U-2 OS cells. Since the viability of CBZ pre-treated OBLs is similar to control OBLs^23^, the pro- or anti-proliferative effects that OBLs exert on OS cells are entirely due to the RANKL/OPG balance. This data shows that CBZ is able to affect OS cells not only in a direct way, but also through its effect on bone microenvironment. In this scenario, RANK expression in OS cells could represent a predictive factor for a better response to CBZ treatment.

**Figure 6:**
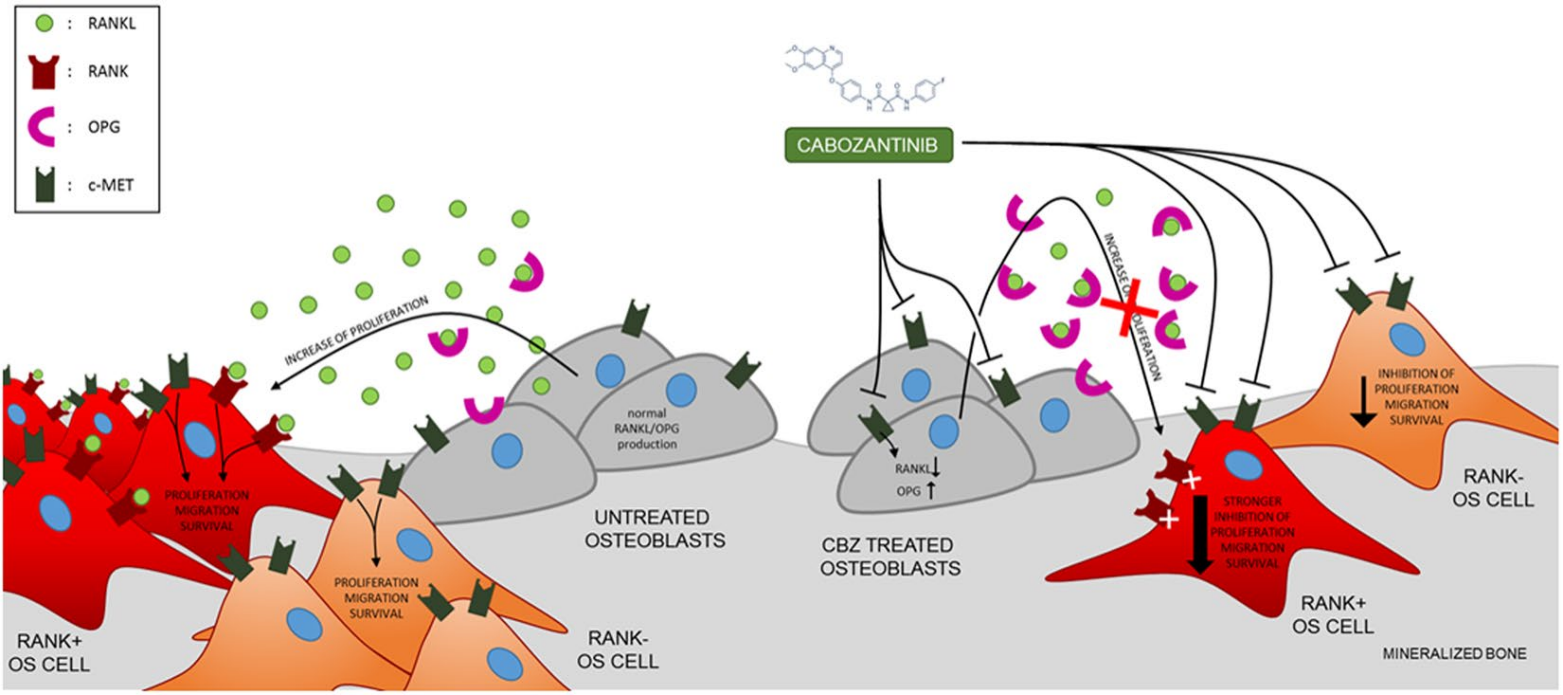
Purposed model of action of CBZ on OS in bone microenvironment. In non treated patients, OS cells can proliferate thanks to c-MET signalling; RANK-positive OS cells could increase their proliferation rate taking advantage of RANKL released from OBLs. In treated patients, CBZ inhibits c-MET pathway and decreases the proliferation of all OS cells; moreover, decreasing RANKL and increasing OPG productions, CBZ causes a stronger inhibition of the proliferation of RANK-positive OS cells.

In summary, OS represents the most common primary malignant tumor of bone and it arises primarily in children and adolescents. Bone microenvironment represents the “soil” where OS can arise and grow, using several bone-related cytokines. High OS heterogeneity and bone microenvironment action lead to the failure of targeted therapies in this tumor setting. The discovery of a new targeted therapy able to inhibit OS through its action on bone microenvironment could improve the therapeutic strategy against OS. Based on our *in vitro* data, we propose a hypothetic novel model of CBZ action in OS and bone microenvironment (Fig. 6). In this highly heterogeneous tumor, c-MET contributes to OS cells transformation and proliferation. Furthermore, in a subset of RANK-positive OS, the proliferation is also stimulated by RANKL produced by OBLs. In light of these data, CBZ might have a double hit effect directly on tumor cell and indirectly on tumor microenvironment reducing the RANKL-induced proliferation. In a treated OS patient, CBZ affects OS cells proliferation through the inhibition of c-MET protein. Moreover, CBZ strongly inhibits the proliferation of RANK-positive OS cells due to the effect on bone microenvironment decreasing the amount of free RANKL that in turn affects osteoclastogenesis blocking the tumor vicious cycle^23^. Thus, considering its whole effects on bone, CBZ could prevent bone lesions that commonly arise in OS patients^2^ inhibiting the production of pro-osteoclastogenic factors by OS cells, leading to a complete improvement of bone health in OS patients. Our hypothesis is only based on data arising from OS cell lines and a simplified bone microenvironment, thus further studies will be useful to confirm the mechanism here proposed. In particular, the complex network of interactions between the tumor and each component of its pathophysiological microenvironment *in vivo* (e.g., different bone cells, immune cells, fibroblasts) could modify the real response of OS to CBZ. Currently, there is an ongoing phase 2 study exploring the activity of CBZ in patients with relapsed OS and Ewing sarcomas (NCT02243605). Based on our results, we suggest to study RANK expression stratifying the patients in order to confirm the possible role of this tyrosine kinase inhibitor as new therapy against OS.

## Materials and Methods

### Cell culture and reagents

HOS, MG-63, Saos-2 and U2OS cell lines were purchased by American Type Culture Collection (ATCC) in 2012. Each cell line was been authenticated by ATCC through morphology, karyotyping, and PCR based approaches. Each cell line was stored in aliquots in liquid nitrogen and used within 6 months once resuscitated. Both HOS and MG-63 cell lines were cultured in RPMI 1640 medium in the presence of 10% heat inactivated fetal bovine serum (FBS). Saos-2 cells were cultured in McCoy medium supplemented with 15% FBS, whereas U-2 OS were cultured in McCoy medium plus 10% FBS. Each culture media were supplemented with 100 units/ml penicillin, 100 mg/ml streptomycin and 2 mM L-glutamine. All cell lines were used between 9^th^ and 18^th^ passage and were periodically tested for mycoplasma infection.

Human mesenchymal stem cells (hMSCs) were isolated from bone fragments of non-oncological orthopedic surgery patients as previously described^23^ and were differentiated in OBLs with alpha MEM (Euroclone) supplemented with 20% FBS (Hyclone, Thermo Scientific), 100 units/ml penicillin, 100 mg/ml streptomycin, amphotericin B (250ng/mL), 10 mM beta-glycerophosphate (Sigma-Aldrich), 50 µM ascorbic acid (Sigma-Aldrich) and 100 nM dexamethasone. CBZ was administrated to mature OBLs from day 21 to day 28 of differentiation protocol every three days.

All OS cell lines and primary human OBLs were grown in 5% CO2 atmosphere at 37 °C.

CBZ was purchased from Selleckchem and was resuspended in dimethyl sulfoxide (DMSO) at a final concentration of 5 mM. Stock solutions were stored at −80 °C and new fresh vial was defrost in each experiment. An appropriate volume of DMSO was supplemented to the negative control.

### Growth curves

In order to evaluate the proliferation of OS cells, 6 × 10^4^ cells were seeded in 3,5 mm plates and allowed to attach overnight; the day after, the cells were treated with 1, 3 and 5 μM of CBZ. The number of cells was assessed daily using a Bürker Chamber after a standard trypsinization. The experiment was repeated three times for each OS cell line.

### Flow cytometry analysis of cell cycle and cell volume

To analyze cell cycle distribution of CBZ treated OS cells, 10^6^ cells for each samples were collected and washed with PBS, fixed dropwise with ice-cold ethanol (70%) and rehydrated with PBS. DNA staining was performed by incubating cells in PBS with 10 μg/mL propidium iodide (PI) and 0.4 mg/ml DNase-free type 1-A RNase overnight at 37 °C. Samples were acquired with a FACSCanto II Flow Cytometer (BDbiosciences). Cell cycle analysis was performed using a FACSDiva software. Cell cycle analysis were repeated in three independent experiments for each OS cell line.

### Time-lapse analysis

OS cells were grown to 70–80% confluency on common petri dishes and treated with different doses of CBZ or DMSO. After 24 hours from the treatment, time-lapse analysis was performed using a Nikon Ti microscope, acquiring images every 20 minutes.

### Wound Healing assay

To analyze the effect of CBZ treatment on migration rate, the OS cells monolayer was scraped in a straight line with a p200 pipet tip. The debris were removed by washing with PBS and then the medium with CBZ or DMSO was replaced. Images of each sample were acquired every 10 hours until the closing of the wound using a Nikon Ti microscope. The evaluation of migration rate was performed analyzing the area between the two sides of the scratch. The experiment was performed three times for each cell line.

### Immunofluorescence

For microscopic analysis of mitotic spindle, OS cells were grown to 70–80% confluency on glass coverslips (Carlo Erba) and treated with different doses of CBZ or DMSO. Samples were fixed using 4% paraformaldehyde for 20 min at 37 °C and then washed with PBS. Permeabilization of cells were performed with 0.1% Triton X-100 for 10 min at room temperature (RT). After a 30 min blocking in 10% Bovine Serum Albumine (BSA), cells were incubated for 1 hour at 37 °C with anti α-tubulin primary antibody (sc53646 SantaCruz, mouse, 1:1000) diluted in PBS. Samples were washed with PBS, and then incubated for 1 hour at RT with AlexaFluor 488-conjugated anti-mouse antibody (Invitrogen, Life Technologies, ab150105, donkey, 1:400) in PBS. Coverslips were washed in PBS and counterstained with 2 μg/mL 4,6-diamidino-2 phenylindole (DAPI) (SigmaAldrich) in Vectashield (Vector Laboratories) antifade. Images of each samples were acquired using a Nikon Ti fluorescence microscope.

### Western Blotting

OS cells were seeded in 50 cm^2^ petri dishes at 50% of confluence and were treated with CBZ 5 μM or DMSO. Cells were scraped at different time points and cell lysates were obtained using radioimmunoprecipitation assay buffer (RIPA buffer) (Sigma-Aldrich) and quantified using DC protein assay kit (Bio-Rad). 30 or 50 μg of the total protein extract from each sample were loaded on 8%/15% SDS-PAGE gels, transferred onto nitrocellulose membranes through Trans- Blot Turbo Transfer System (Bio-Rad). In order to block the non-specific sites, membranes were incubated in a blocking buffer (TBST 1 × with 5% non-fat dry milk) for one hour. Membranes were incubated with primary antibody overnight shacking at 4 °C. Anti-β-Actina (A2228, mouse, 1:10000) and anti-LC3 (L7543, rabbit, 1:1000) antibodies were purchased from Sigma Aldrich; anti-c-Met (rabbit 8198), anti-phospho c-Met Tyr1234/1235 (3077, rabbit, 1:1000), anti-p44/42 ERK1/2 (4695, rabbit, 1:1000), anti-phospho p44/42 ERK1/2 Thr202/Tyr204 (4370, rabbit, 1:2000), anti-AKT pan (4691, rabbit, 1:1000) and anti-phospho AKT Thr308 (13038, rabbit, 1:1000) antibodies were purchased from Cell Signaling; anti-ULK1 (SC33182, rabbit, 1:1000) and anti-Beclin 1 (SC11427, rabbit, 1:1000) antibodies were purchased from SantaCruz and anti-RANK (ab182158, rabbit, 1:1000) antibody was purchased from Abcam. Anti-rabbit/mouse HRT-coniugated antibody (Abcam) (1:10000 diluted) was used and the chemiluminescence signal detected using ChemiDoc (Bio-Rad) and Quantity One software (Bio-Rad) to quantify the signal intensity of different bands.

### GFP stable transfection

Constitutive expression of Green Fluorescent Protein (GFP) was achieved by stable transfection of OS cells using MISSION® pLKO.1-puro-CMV-TurboGFP™ Positive Control Transduction Particles (Sigma Aldrich). In particular, OS cells were seeded in 96-multi well plate at 70–80% of confluence. After 12 hours the culture media was removed and replaced with fresh culture media plus 8 mg/mL of Hexadimethrine bromide and lentiviral particles using a 0,5 of multiplicity of infection (MOI). 2 μg/mL of Puromycin was added after 48 hours in order to select the transfected GFP + cells.

### Co-culture experiments

OBLs were treated with different doses of CBZ or DMSO as previously described^23^. After the end of the treatment each sample was washed three times with PBS and 2500 GFP + OS cells/cm^2^ were seeded directly on OBL layer, with no drug replacing. Images of each sample were acquired after 6 hours from the seeding (t0) in order to allow the cell attachment, and every 24 hours, using a Nikon Ti fluorescence microscope. The number of GFP + OS cells where counted for each time point.

### Statistical analysis

Data were analyzed using the Student t test and One-Way ANOVA test followed by Tukey’s multiple comparison tests. The graphics processing and statistical tests were performed using the program GraphPad Prism (San Diego, CA). P-values lower than 0.05 were considered significant.

## Electronic supplementary material

Supplementary Data

## References

[1] Yang J, Zhang W (2013). New molecular insights into osteosarcoma targeted therapy. Curr. Opin. Oncol..

[2] Kansara M, Teng MW, Smyth MJ, Thomas DM (2014). Translational biology of osteosarcoma. Nat. Rev. Cancer.

[3] Whelan JS (2015). EURAMOS-1, an international randomised study for osteosarcoma: results from pre-randomisation treatment. Ann. Oncol..

[4] Bielack SS (2015). Methotrexate, Doxorubicin, and Cisplatin (MAP) Plus Maintenance Pegylated Interferon Alfa-2b Versus MAP Alone in Patients With Resectable High-Grade Osteosarcoma and Good Histologic Response to Preoperative MAP: First Results of the EURAMOS-1 Good Response Randomized Controlled Trial. J. Clin. Oncol..

[5] Messerschmitt PJ (2008). Specific tyrosine kinase inhibitors regulate human osteosarcoma cells *in vitro*. Clin. Orthop. Relat. Res..

[6] MacEwen EG (2003). c-Met tyrosine kinase receptor expression and function in human and canine osteosarcoma cells. Clin. Exp. Metastasis.

[7] Pignochino Y (2009). Sorafenib blocks tumour growth, angiogenesis and metastatic potential in preclinical models of osteosarcoma through a mechanism potentially involving the inhibition of ERK1/2, MCL-1 and ezrin pathways. Mol. Cancer.

[8] Pignochino Y (2013). The Combination of Sorafenib and Everolimus Abrogates mTORC1 and mTORC2 upregulation in osteosarcoma preclinical models. Clin. Cancer Res..

[9] Kumar RM, Arlt MJ, Kuzmanov A, Born W, Fuchs B (2015). Sunitinib malate (SU-11248) reduces tumour burden and lung metastasis in an intratibial human xenograft osteosarcoma mouse model. Am. J. Cancer Res..

[10] Ehnman M, Larsson O (2015). Microenvironmental Targets in Sarcoma. Front. Oncol..

[11] Alfranca A (2015). Bone microenvironment signals in osteosarcoma development. Cell. Mol. Life Sci..

[12] Mori K (2007). Human osteosarcoma cells express functional receptor activator of nuclear factor-kappa B. J. Pathol..

[13] Bago-Horvath Z (2014). Impact of RANK signalling on survival and chemotherapy response in osteosarcoma. Pathology.

[14] Viola D, Cappagli V, Elisei R (2013). Cabozantinib (XL184) for the treatment of locally advanced or metastatic progressive medullary thyroid cancer. Future Oncol..

[15] Smith DC (2013). Cabozantinib in patients with advanced prostate cancer: results of a phase II randomized discontinuation trial. J. Clin. Oncol..

[16] Choueiri TK (2015). Cabozantinib versus Everolimus in Advanced Renal-Cell Carcinoma. N. Engl. J. Med..

[17] Patanè S (2006). MET overexpression turns human primary osteoblasts into osteosarcomas. Cancer Res..

[18] Dani N (2012). The MET oncogene transforms human primary bone-derived cells into osteosarcomas by targeting committed osteo-progenitors. J. Bone Miner. Res..

[19] Sampson ER (2011). The orally bioavailable met inhibitor PF-2341066 inhibits osteosarcoma growth and osteolysis/matrix production in a xenograft model. J. Bone Miner. Res..

[20] Dai J (2014). Cabozantinib inhibits prostate cancer growth and prevents tumor-induced bone lesions. Clin. Cancer Res..

[21] Stern PH, Alvares K (2014). Antitumor agent cabozantinib decreases RANKL expression in osteoblastic cells and inhibits osteoclastogenesis and PTHrP-stimulated bone resorption. J. Cell. Biochem..

[22] Haider MT (2015). Rapid modification of the bone microenvironment following short-term treatment with Cabozantinib *in vivo*. Bone.

[23] Fioramonti M (2017). Cabozantinib targets bone microenvironment modulating human osteoclast and osteoblast functions. Oncotarget.

[24] Mizushima N, Yoshimori T, Levine B (2010). Methods in mammalian autophagy research. Cell.

[25] Organ SL, Tsao MS (2011). An overview of the c-MET signalling pathway. Ther. Adv. Med. Oncol..

[26] Cooper CS (1984). Molecular cloning of a new transforming gene from a chemically transformed human cell line. Nature.

[27] Scotlandi K (1996). Expression of Met/hepatocyte growth factor receptor gene and malignant behavior of musculoskeletal tumors. Am. J. Pathol..

[28] Yakes FM (2011). Cabozantinib (XL184), a novel MET and VEGFR2 inhibitor, simultaneously suppresses metastasis, angiogenesis, and tumor growth. Mol. Cancer Ther..

[29] Wang X (2015). Cabozantinib Inhibits Abiraterone’s Upregulation of IGFIR Phosphorylation and Enhances Its Anti-Prostate Cancer Activity. Clin. Cancer Res..

[30] Xie Z (2016). MET Inhibition in Clear Cell Renal Cell Carcinoma. J. Cancer.

[31] Neradil J (2015). DHFR-mediated effects of methotrexate in medulloblastoma and osteosarcoma cells: the same outcome of treatment with different doses in sensitive cell lines. Oncol. Rep..

[32] Shor AC (2007). Dasatinib inhibits migration and invasion in diverse human sarcoma cell lines and induces apoptosis in bone sarcoma cells dependent on SRC kinase for survival. Cancer Res..

[33] Sui X (2013). Autophagy and chemotherapy resistance: a promising therapeutic target for cancer treatment. Cell Death Dis..

