# Supplementary material for: Cabozantinib Affects Osteosarcoma Growth Through A Direct Effect On Tumor Cells and Modifications In Bone Microenvironment
Source: Sci Rep. 2018 Mar 8;8:4177. doi: 10.1038/s41598-018-22469-5 (PMC5843583; doi:10.1038/s41598-018-22469-5)
Supplement: Supplementary file 1 — Supplementary Data [file 41598_2018_22469_MOESM1_ESM.pdf]

Cabozantinib Affects Osteosarcoma Growth Through A Direct Effect On Tumor Cells And Modifications In Bone Microenvironment

Fioramonti M[1*], Fausti V[2,3], Pantano F[1], Iuliani M[1], Ribelli G[1], Lotti F[4], Pignochino Y[5], Grignani G[5], Santini D[1], Tonini G[1], Vincenzi B[1]

1: Medical Oncology, Campus Bio-Medico University of Rome, Rome, Italy

2: Medical Oncology, University of Ferrara, Ferrara, Italy

3: Osteoncology and Rare Tumors Center, Istituto Scientifico Romagnolo per lo Studio e la Cura dei Tumori (IRST) IRCCS, Meldola (FC), Italy

4: Orthopaedics and Trauma Surgery, Campus Bio-Medico University of Rome, Rome, Italy

5: Sarcoma Unit, Division of Medical Oncology, Candiolo Cancer Institute-FPO, IRCCS, Candiolo (TO), Italy

*corresponding author: Medical Oncology, Campus Bio-Medico University of Rome, via Alvaro del Portillo 200, 00128, Rome, Italy Tel: +39 06225419117 - Fax: +39 06225411933 – email: ma.fioramonti@gmail.com

SUPPLEMENTARY DATA

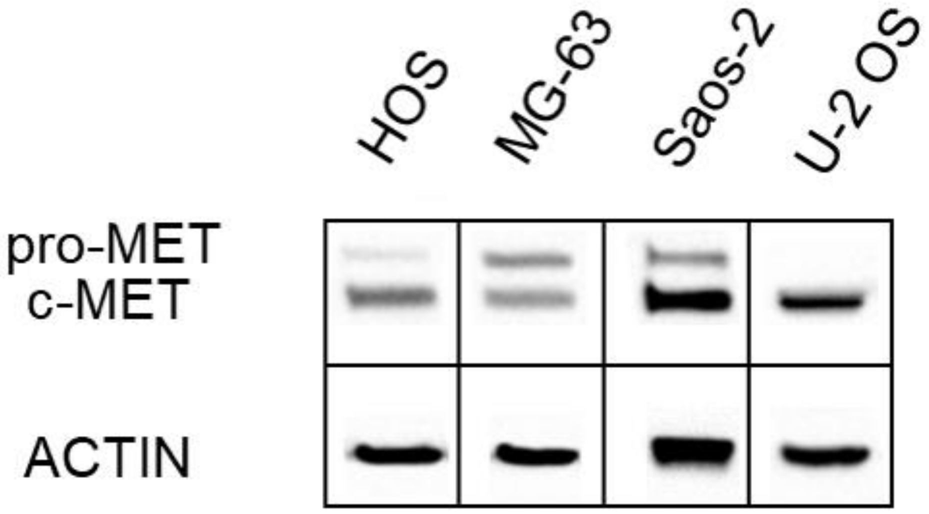

Figure 1 suppl. data: Western blot analysis of the expression of c-MET receptor in HOS, MG-63, Saos-2 and U-2 OS cells. Inactive form of the receptor, named pro-MET (~175 kDa), is not detectable in U-2 OS cell line only. The active c-MET receptor levels (~140 kDa) are similar in each tested OS cell line, compared to actin levels. Full length blots are presented in supplementary figure 4 (a-d) and are the same used for figure 4.

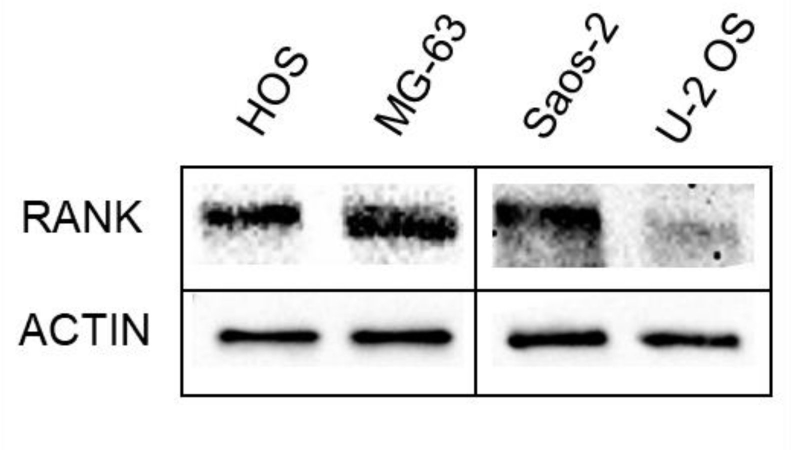

Figure 2 suppl. data: Evaluation of the expression of RANK in OS cells; HOS, MG-63 and Saos-2 cells are positive to RANK expression, whereas U-2 OS cells do not express the receptor. Full length blots are showed in supplementary figure 4 e.

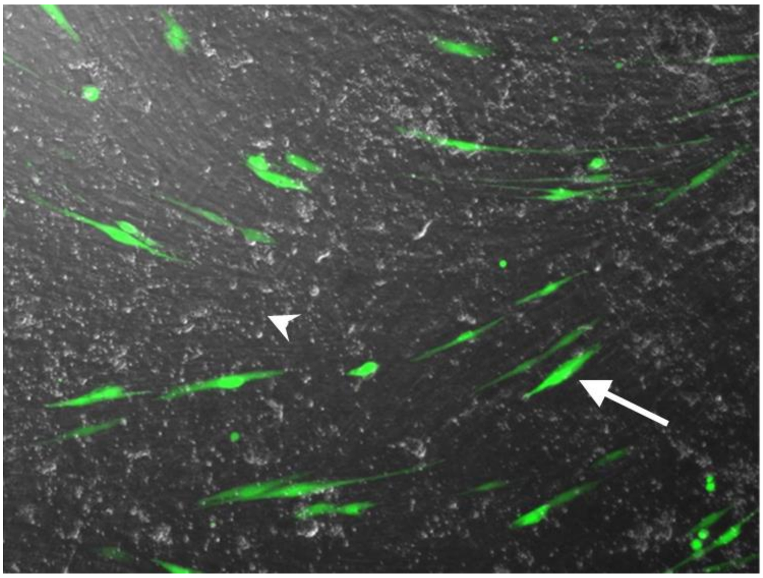

Figure 3 suppl. data: representative image of co-culture between OS cells and OBLs. White arrow indicates GFP+ OS cells; arrow-head indicates OBLs cells with their typical calcium deposits.

a)

HOS WESTERN BLOTS

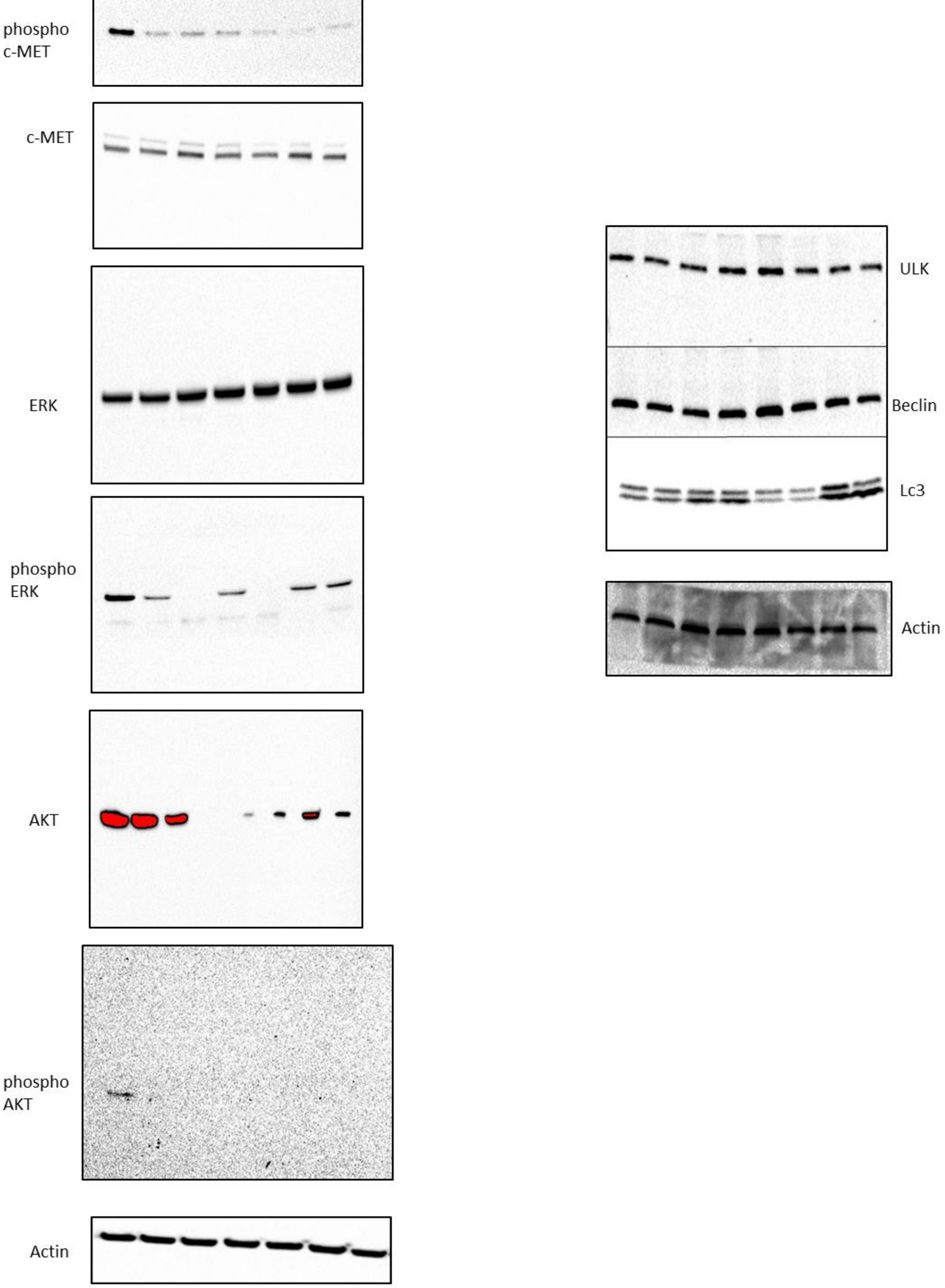

b)

MG-63 WESTERN BLOTS

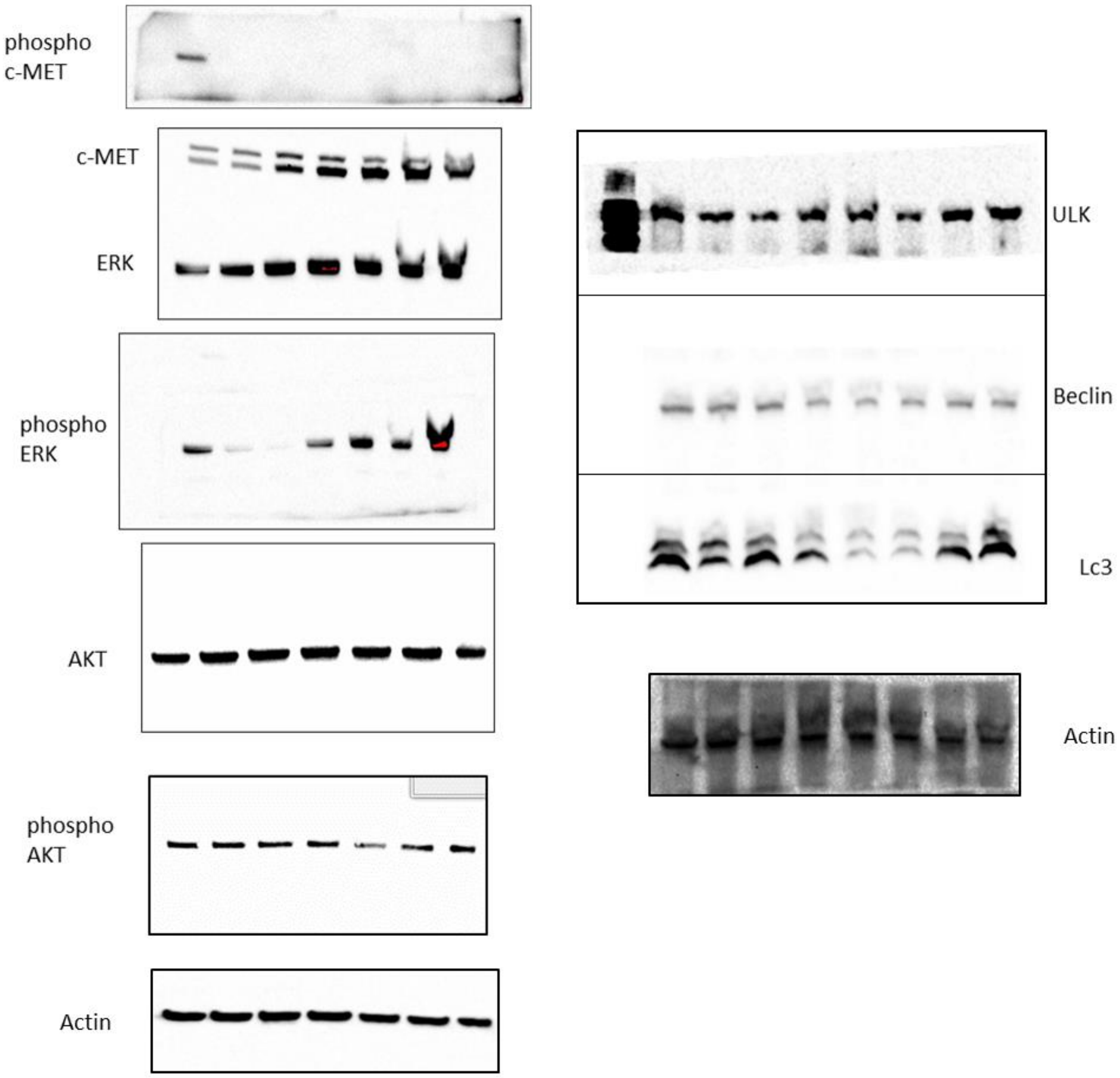

c)

Saos-2 WESTERN BLOTS

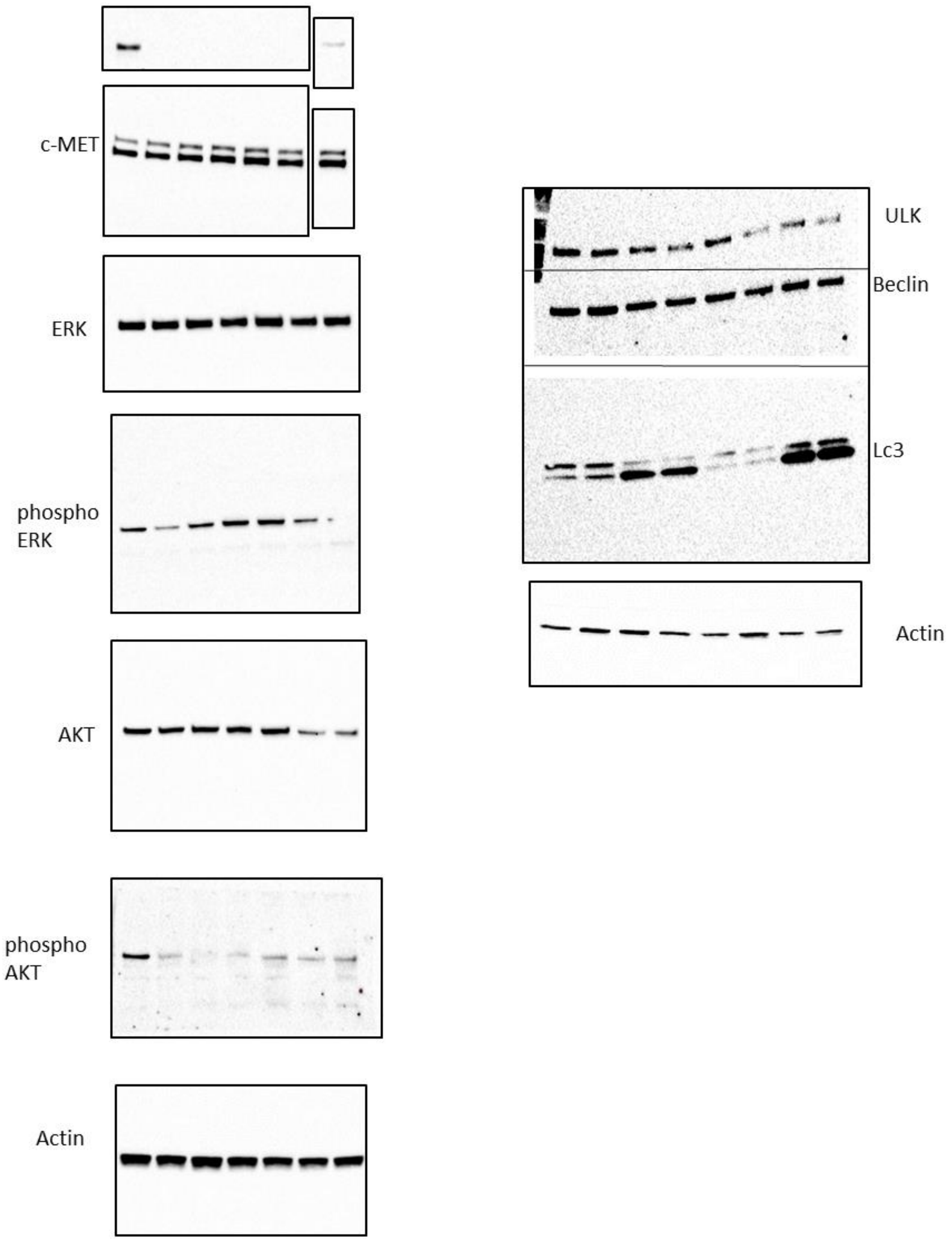

d)

U-2 OS WESTERN BLOTS

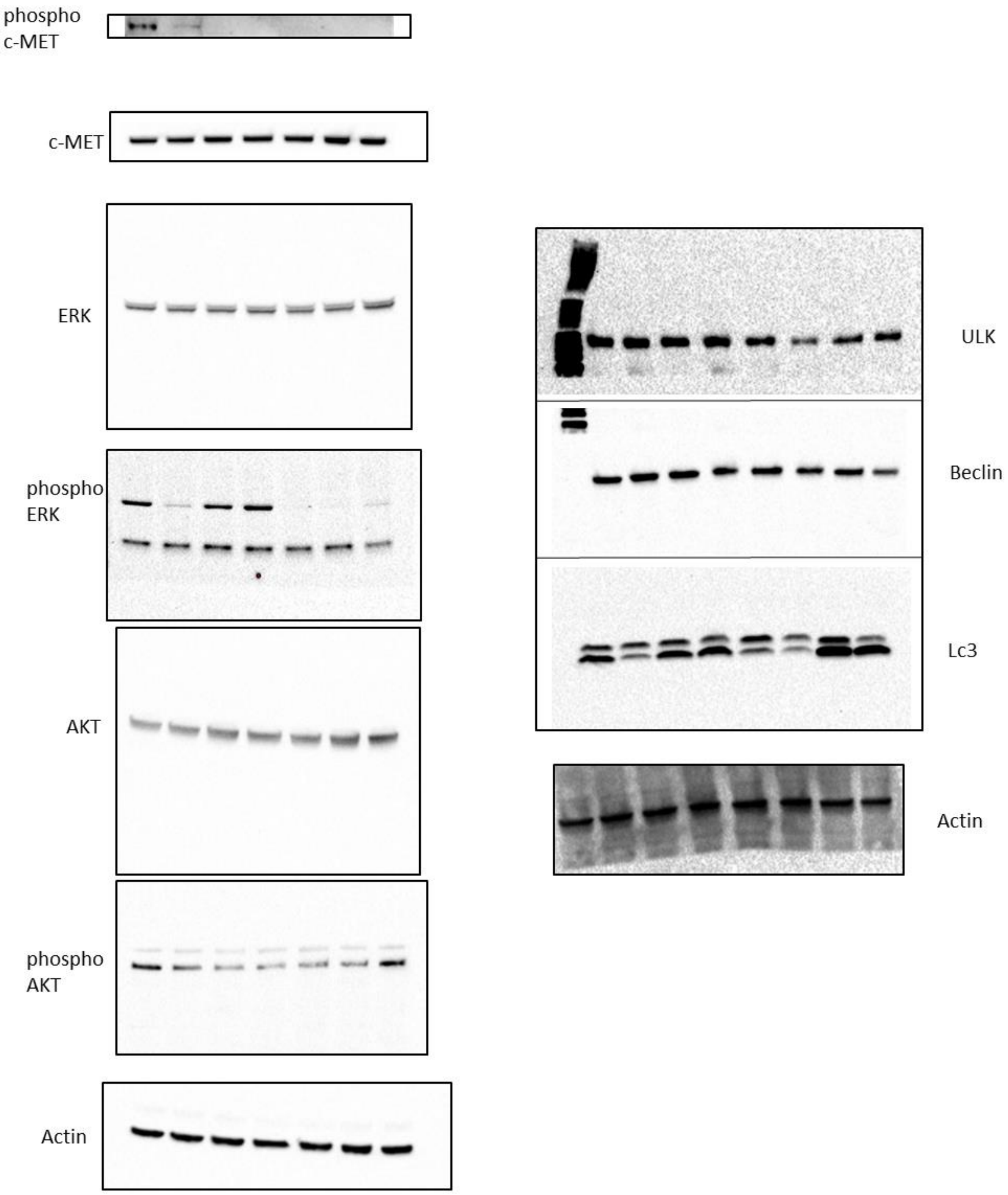

e)

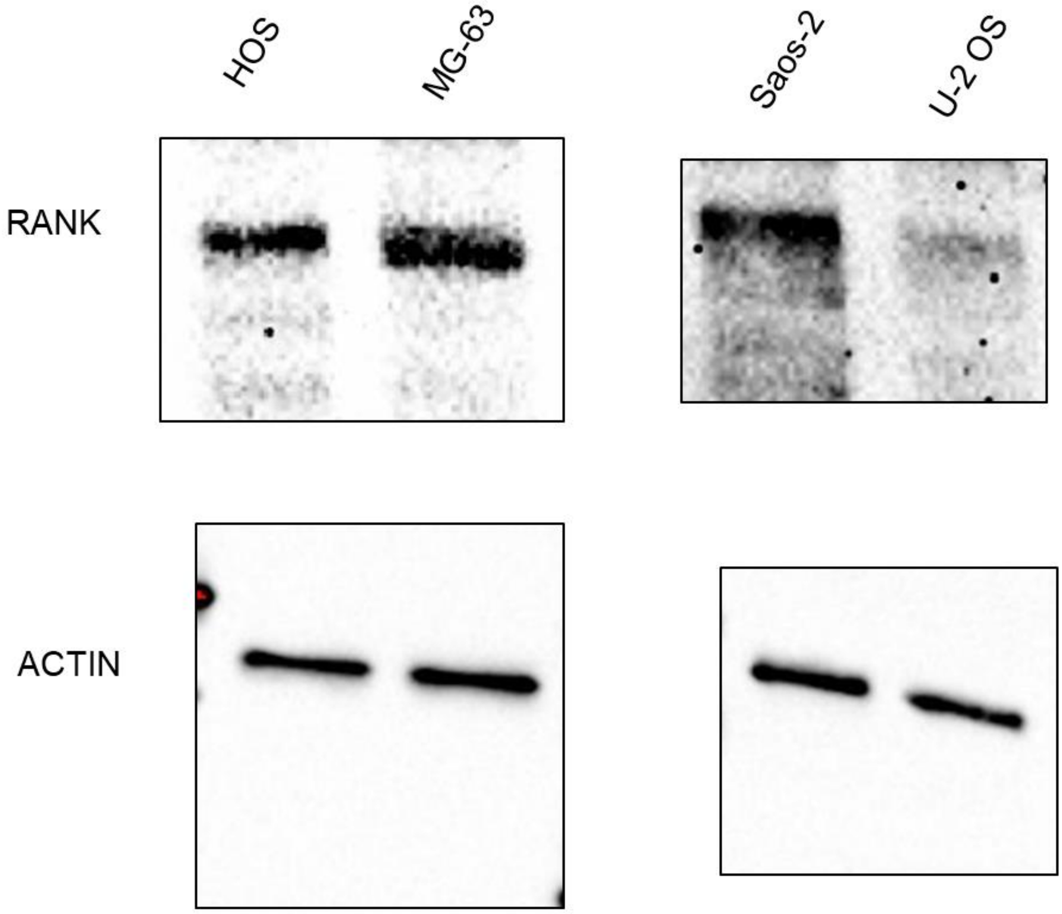

Figure 4 suppl. data: full length blots of figure 4/supplementary figure 1 (a, b, c, d) and supplementary figure 2 (e).
